# Supplementary material for: Synergistic Anticancer Effects of Fibroblast Growth Factor Receptor Inhibitor and Cannabidiol in Colorectal Cancer
Source: Nutrients. 2025 Aug 12;17(16):2609. doi: 10.3390/nu17162609 (PMC12389608; doi:10.3390/nu17162609)
Supplement: Supplementary file 1 [file nutrients-17-02609-s001.zip › nutrients-3759232-supplementary.pdf]

Supplementary Materials:

Supplementary Figure S1: Effects of other FGFR inhibitors (PD173074 and BGJ398) on proliferation and FGFR signaling in NCI-H716 colorectal cancer cells.

(A) Dose–response curve showing the effect of PD173074 treatment (1–10,000 nM) for 72 h on NCI-H716 cell proliferation, measured by WST-1 assay. Data are presented as mean ± SD from three independent experiments.

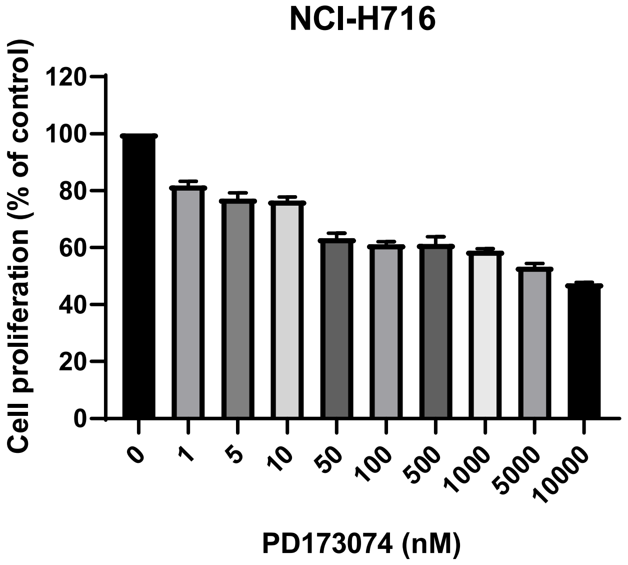

(B) Western blot analysis of NCI-H716 cells treated with PD173074 (10 nM) for 24 h, showing inhibition of FGFR phosphorylation and downstream signaling pathways (AKT, ERK, STAT3). β-actin was used as a loading control.

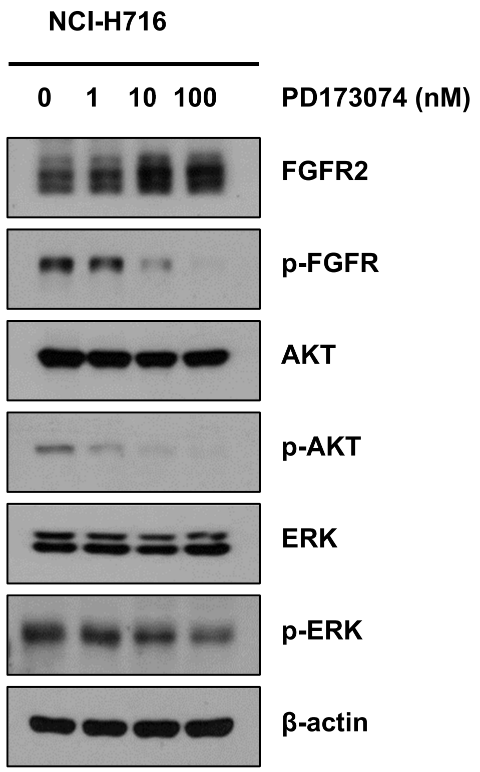

(C) Dose–response curve showing the effect of BGJ398 treatment (1–10,000 nM) for 72 h on NCI-H716 cell proliferation, measured by WST-1 assay. Data are presented as mean ± SD from three independent experiments.

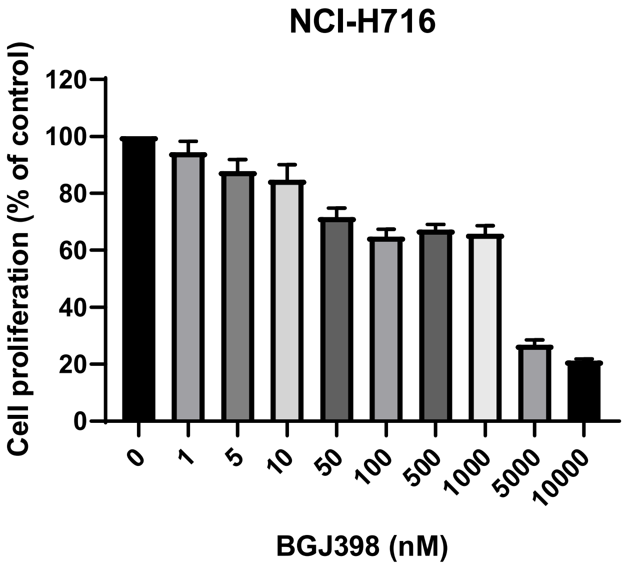

(D) Western blot analysis of NCI-H716 cells treated with BGJ398 (10 nM) for 24 h, showing inhibition of FGFR phosphorylation and downstream signaling pathways (AKT, ERK, STAT3). β-actin was used as a loading control.

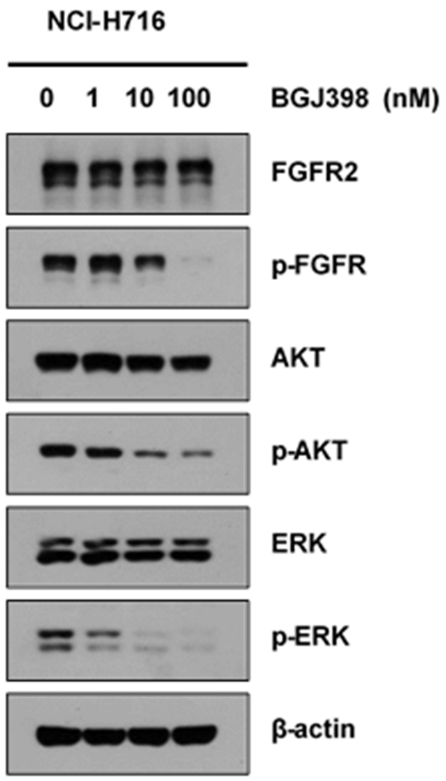

Supplementary Figure S2: Effects of CBD in combination with FGFR inhibitors on apoptosis-related protein expression and apoptosis induction in colorectal cancer cell lines.

(A) Western blot analysis of apoptosis-related proteins in FGFR-low colorectal cancer cell lines (HCT116, HT29, DLD-1) treated with CBD (4 μM), AZD4547 (10 nM), or their combination for 24 h. β-actin was used as a loading control.

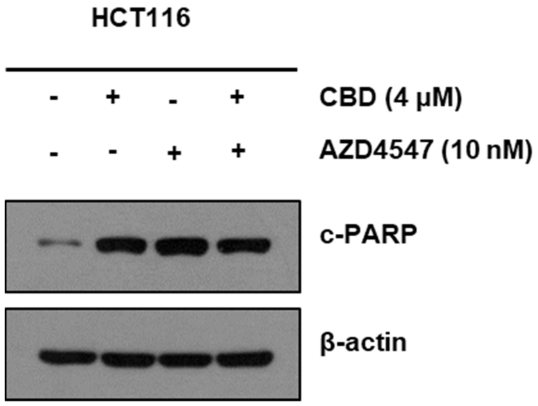

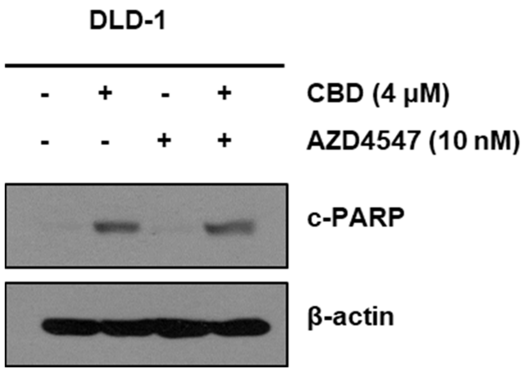

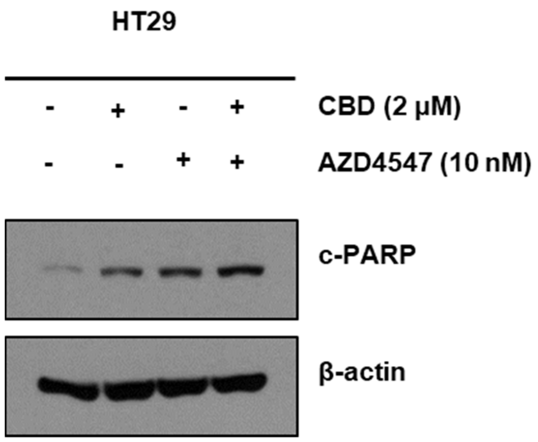

(B) Annexin V-FITC/PI staining of NCI-H716 cells treated for 24 h with CBD (4 μM), PD173074 (10 nM), or their combination (CBD + PD173074), compared with vehicle control.

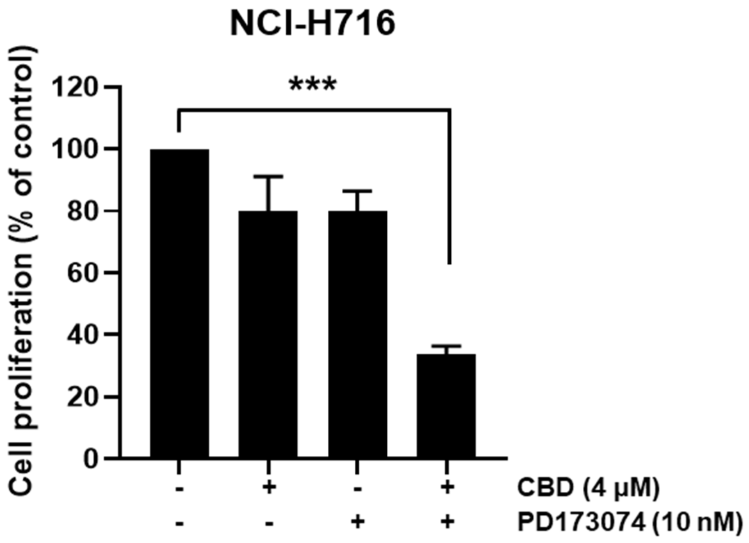

(C) Annexin V-FITC/PI staining of NCI-H716 cells treated for 24 h with CBD (4 μM), BGJ398 (10 nM), or their combination (CBD + BGJ398), compared with vehicle control.

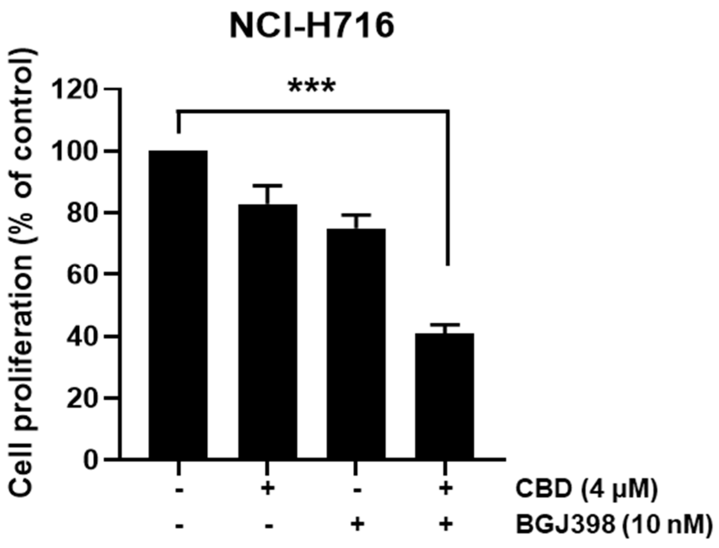

Supplementary Figure S3: Differential gene expression profiles and validation plots from RNA-seq analysis of NCI-H716 cells under different treatment conditions.

(A) Heatmap showing the top differentially expressed genes at the gene level among control (Ct), AZD4547 (AA), CBD (CC), and CBD + AZD4547 (AC) treatment groups.

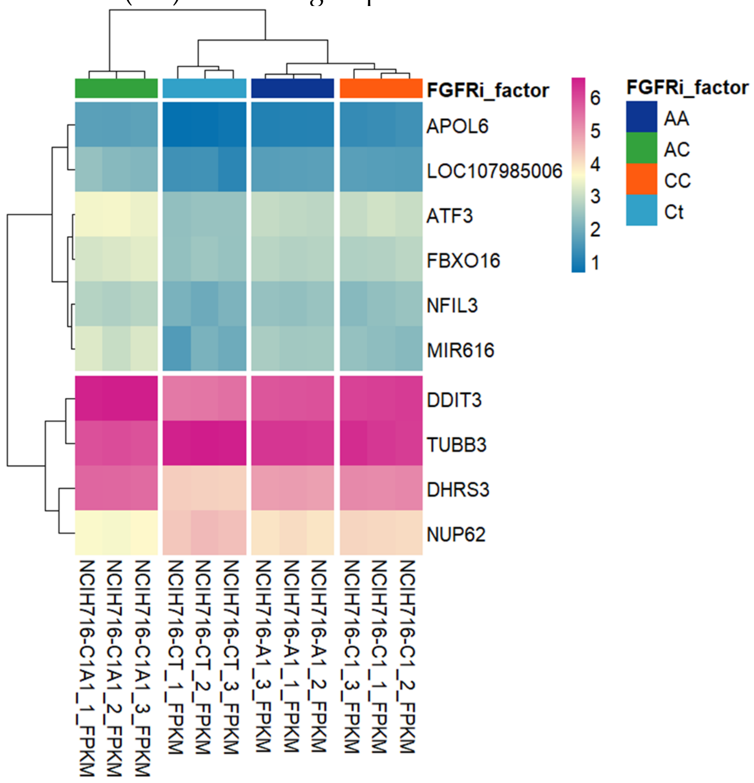

(B) Heatmap showing the top differentially expressed genes at the transcript level among the same treatment groups.

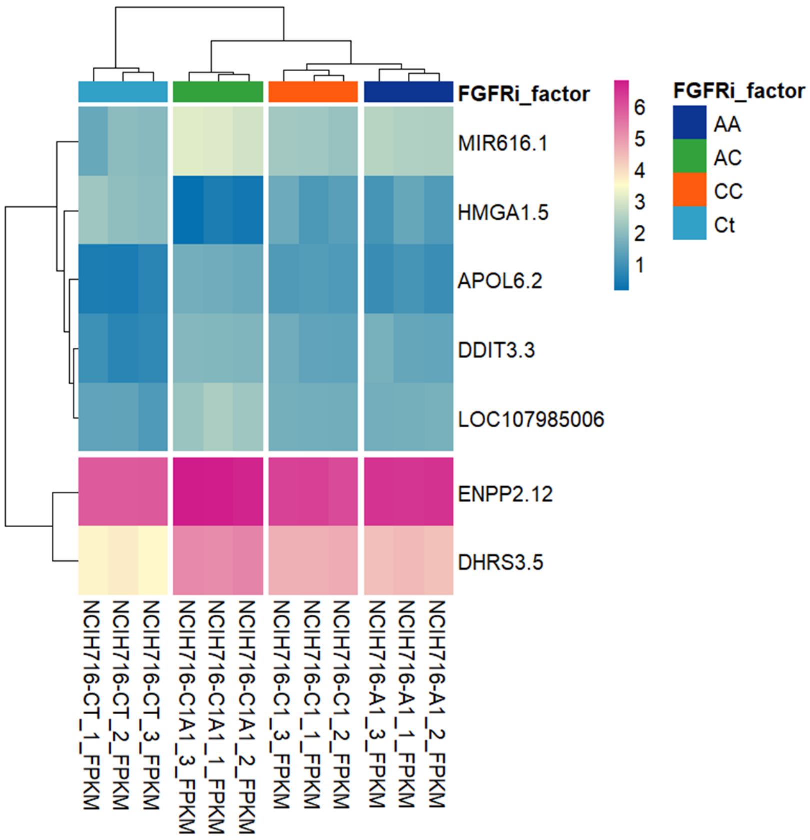

(C) Boxplots showing normalized expression levels (FPKM) of the top differentially expressed genes from (A), including ATF3, DDIT3, NFIL3, DHRS3, APOL6, FBXO16, MIR616, LOC107985006, TUBB3, and NUP62.

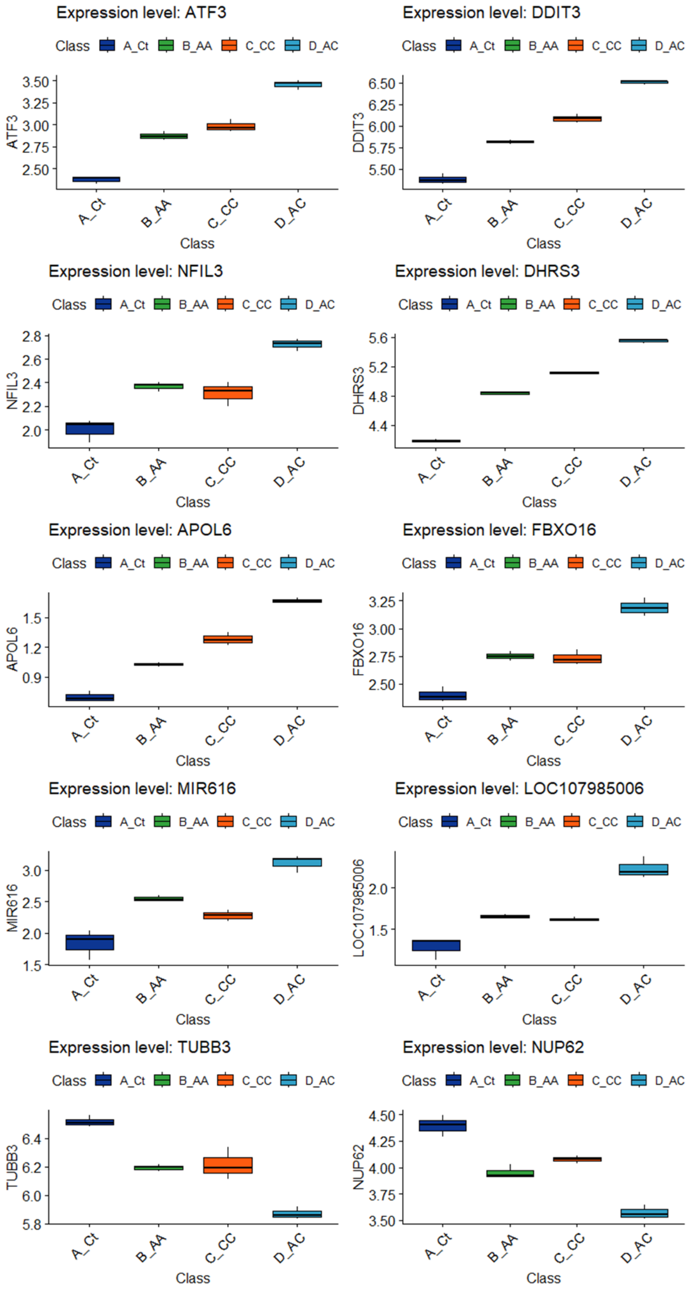

(D) Boxplots showing normalized expression levels (FPKM) of the top differentially expressed transcripts from (B), including DDIT3.3, ENPP2.12, DHRS3.5, APOL6.2, MIR616.1, LOC107985006, and HMGA1.5.

H

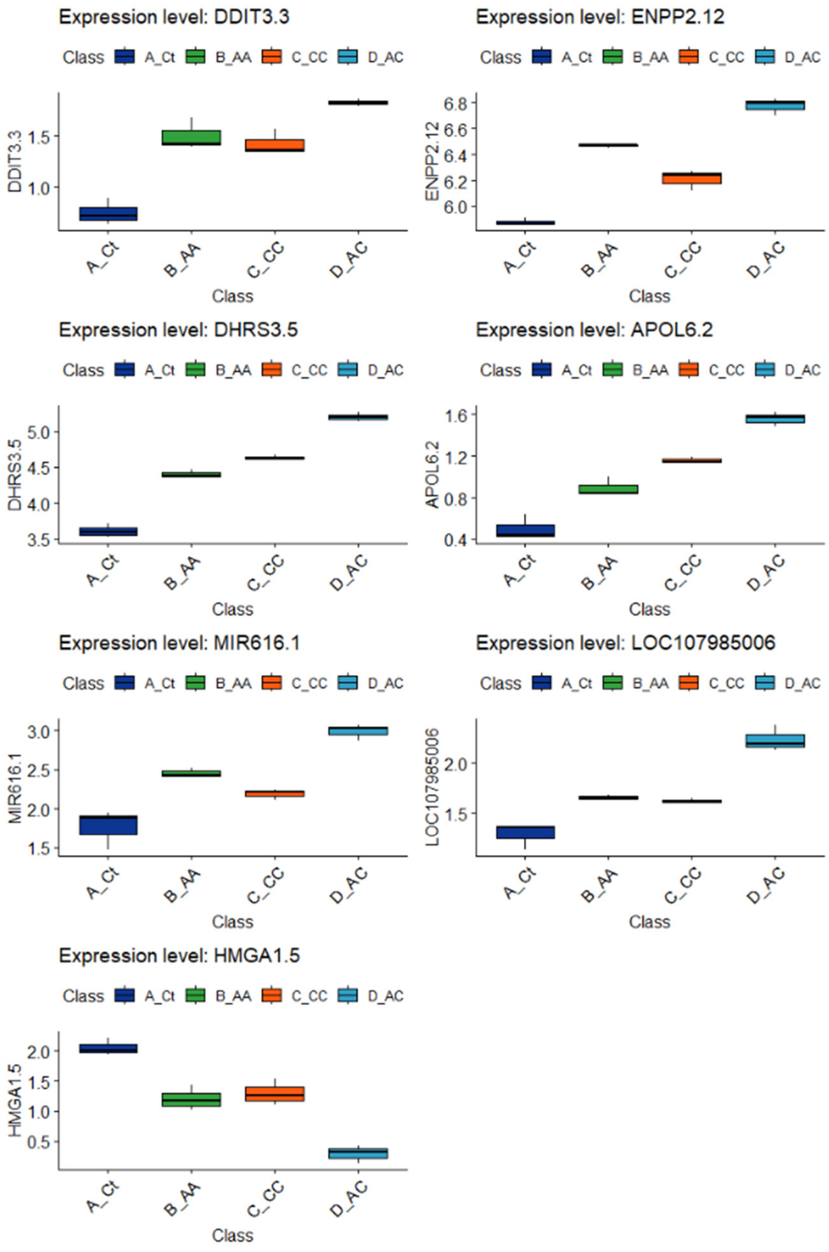

Expression level: DDIT3.3
Class A_Ct B_AA C_CC D_AC
DDIT3.3
1.0
1.5
A_Ct B_AA C_CC D_AC
Class
Expression level: ENPP2.12
Class A_Ct B_AA C_CC D_AC
ENPP2.12
6.0
6.2
6.4
6.6
6.8
A_Ct B_AA C_CC D_AC
Class
Expression level: DHRS3.5
Class A_Ct B_AA C_CC D_AC
DHRS3.5
3.5
4.0
4.5
5.0
A_Ct B_AA C_CC D_AC
Class
Expression level: APOL6.2
Class A_Ct B_AA C_CC D_AC
APOL6.2
0.4
0.8
1.2
1.6
A_Ct B_AA C_CC D_AC
Class
Expression level: MIR616.1
Class A_Ct B_AA C_CC D_AC
MIR616.1
1.5
2.0
2.5
3.0
A_Ct B_AA C_CC D_AC
Class
Expression level: LOC107985006
Class A_Ct B_AA C_CC D_AC
LOC107985006
1.5
2.0
A_Ct B_AA C_CC D_AC
Class
Expression level: HMGA1.5
Class A_Ct B_AA C_CC D_AC
HMGA1.5
0.5
1.0
1.5
2.0
A_Ct B_AA C_CC D_AC
Class
